# Ferritinophagy: multifaceted roles and potential therapeutic strategies in liver diseases

**DOI:** 10.3389/fcell.2025.1551003

**Published:** 2025-02-25

**Authors:** Kejia Wu, Wei Zhao, Zeyu Hou, Weigang Zhang, Lei Qin, Junyi Qiu, Daobin Wang, Lin Zhuang, Xiaofeng Xue, Ding Sun

**Affiliations:** ^1^ Department of Hepatopancreatobiliary Surgery, The First Affiliated Hospital of Soochow University, Suzhou, China; ^2^ Department of Anesthesiology, Xinyi People’s Hospital, Xinyi, Jiangsu, China; ^3^ Department of General Surgery, Wujin Affiliated Hospital of Jiangsu University and The Wujin Clinical College of Xuzhou Medical University, Changzhou, Jiangsu, China

**Keywords:** ferritinophagy, autophagy, ferroptosis, NAFLD, HCC, liver fibrosis

## Abstract

Ferritinophagy, the selective autophagic degradation of ferritin to release iron, is emerging as a critical regulator of iron homeostasis and a key player in the pathogenesis of various liver diseases. This review comprehensively examines the mechanisms, regulation, and multifaceted roles of ferritinophagy in liver health and disease. Ferritinophagy is intricately regulated by several factors, including Nuclear Receptor Coactivator 4 (NCOA4), Iron regulatory proteins and signaling pathways such as mTOR and AMPK. These regulatory mechanisms ensure proper iron utilization and prevent iron overload, which can induce oxidative stress and ferroptosis. In liver diseases, ferritinophagy exhibits dual roles. In liver fibrosis, promoting ferritinophagy in hepatic stellate cells (HSCs) can induce cell senescence and reduce fibrosis progression. However, in non-alcoholic fatty liver disease (NAFLD), chronic ferritinophagy may exacerbate liver injury through iron overload and oxidative stress. In hepatocellular carcinoma (HCC), ferritinophagy can be harnessed as a novel therapeutic strategy by inducing ferroptosis in cancer cells. Additionally, ferritinophagy is implicated in drug-induced liver injury and sepsis-associated liver damage, highlighting its broad impact on liver pathology. This review also explores the crosstalk between ferritinophagy and other selective autophagy pathways, such as mitophagy and lipophagy, which collectively influence cellular homeostasis and disease progression. Understanding these interactions is essential for developing comprehensive therapeutic strategies targeting multiple autophagy pathways. In summary, ferritinophagy is a complex and dynamic process with significant implications for liver diseases. This review provides an in-depth analysis of ferritinophagy’s regulatory mechanisms and its potential as a therapeutic target, emphasizing the need for further research to elucidate its role in liver health and disease.

## Introduction

The phenomenon of autophagy was first discovered by scholars Ashford and Porter in 1962. However, at that time, they interpreted their observations as the process of lysosome formation and believed that lysosomes were not independent organelles but rather a part of mitochondria (Ashford and Porter, 1962). It was not until 1967 that Duve and his colleagues formally named this phenomenon “autophagy” and identified lysosomes as the sites where autophagy occurs (Deter et al., 1967). Over the next 30 years, numerous researchers conducted studies based on this foundation, discovering and naming several autophagy-related proteins. To minimize confusion and complications arising from different naming conventions, a unified gene and protein nomenclature was established (Klionsky et al., 2003).

Autophagy is an intracellular degradation and recycling pathway. It involves encapsulating target substances into autophagosomes, which are then sent to lysosomes for degradation. This process, which helps to mitigate damage caused by metabolic stress and clear out unnecessary or harmful substances within the cell, plays a crucial role in maintaining cellular homeostasis (Patel et al., 2013). Ferroptosis, another emerging research hotspot, was initially regarded as a novel form of cell death dependent on intracellular iron. Unlike traditional apoptosis, autophagy, and necrosis, ferroptosis is primarily caused by the excessive accumulation of iron ions and lipid peroxidation within cells (Dixon et al., 2012). However, in recent years, researchers have discovered a close link between autophagy and ferroptosis. The most representative example is ferritinophagy, which involves the degradation of ferritin to release free Fe2+ ions. These Fe2+ ions generate excessive ROS through the Fenton reaction, thereby inducing ferroptosis (Qian et al., 2024).

Ferritin, the primary iron storage protein in the human body, is crucial for maintaining iron homeostasis. It regulates intracellular iron balance by storing and releasing iron as needed (Knovich et al., 2009). Structurally, ferritin is a 24-subunit macromolecular iron-storage complex composed of ferritin heavy chain (FTH1) and ferritin light chain (FTL) subunits. FTH1 can oxidize ferrous iron (Fe^2^⁺) to ferric iron (Fe^3^⁺), allowing it to be safely stored within the ferritin complex. While FTL promotes iron nucleation, enhancing the stability of the ferritin structure (Pantopoulos et al., 2012). As early as 2005, Theodros and his colleagues discovered that lysosomes play a crucial role in degrading ferritin and releasing iron ions (Kidane et al., 2006). But It was not until 2014 that a paper published in Nature demonstrated that NCOA4 is a key receptor for ferritinophagy. NCOA4 mediates the localization of ferritin to autophagosomes, which then fuse with lysosomes to complete the degradation process. (Mancias et al., 2014). Under normal circumstances, ferritinophagy adjusts according to intracellular iron levels. When there is an excess of iron, NCOA4 can be ubiquitinated and degraded, promoting the storage of surplus iron and preventing its toxic effects. In conditions of iron deficiency, NCOA4 selectively binds to ferritin, facilitating its degradation through autophagy to release iron, thereby maintaining normal cellular iron levels and physiological activities (Mancias et al., 2015; Wu H. et al., 2023).

Recent research has found that ferritinophagy is not only crucial for maintaining iron balance under normal physiological conditions but also closely related to the development and progression of various diseases. Under conditions of toxicity or oxidative stress, overexpression of ferritinophagy can lead to a range of liver diseases. For example, exposure to metals and chemicals such as cadmium, copper, and arsenic (As) can induce ferritinophagy and result in liver damage (He et al., 2022; Yu L. et al., 2023; Zhong et al., 2024). In patients with NAFLD, short-term ferritinophagy can reduce lipotoxicity. However, chronic ferritinophagy often indicates worsening of the condition, progressing from reversible fatty liver to irreversible liver cirrhosis (Li et al., 2022). From another perspective, harnessing ferritinophagy to target specific detrimental factors can also benefit certain diseases. Such as, in liver fibrosis, employing specific drugs to enhance ferritinophagy in HSCs can effectively mitigate the progression of liver fibrosis (Kong et al., 2019). In this context, this review aims to summarize the fundamental mechanisms and regulatory pathways of ferritinophagy, along with its potential roles in various diseases. The goal is to offer new insights and advance research in this field.

### Regulation of ferritinophagy

**FIGURE 1:**
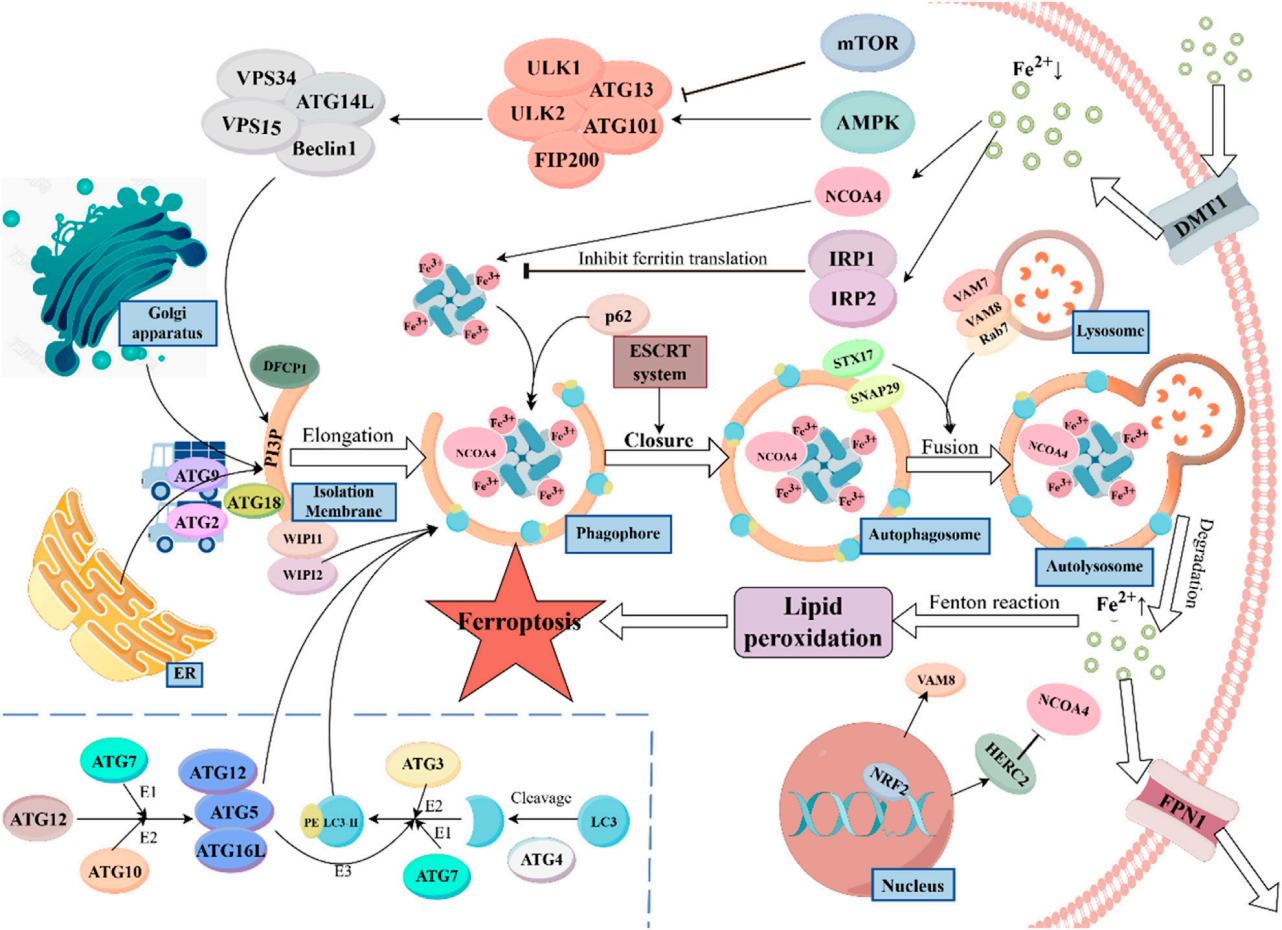
Ferritinophagy, a specific form of autophagy, is vital for maintaining cellular iron homeostasis. The process begins with the recognition of ferritin by Nuclear Receptor Coactivator 4 (NCOA4), which directs ferritin to the isolation membrane (IM). Upon autophagosome maturation, ferritin is degraded in the lysosome, releasing iron ions, which are exported by Ferroportin 1 (FPN1). Divalent Metal Transporter 1 (DMT1) mediates iron uptake through endosomal formation. The autophagic process is regulated by mechanistic target of rapamycin (mTOR) and AMP-activated protein kinase (AMPK) signaling in response to nutrient availability. The Autophagy-Related Gene 16-Like (ATG16L) complex facilitates the lipidation of Light Chain 3 (LC3), essential for autophagosome formation. Transport proteins such as ATG9, ATG2, and ATG18 are involved in lipid transfer, supporting the expansion of the autophagosome. Additionally, the protein WD Repeat Domain Phosphoinositide Interacting Proteins 1/2 (WIPI1/2) plays a crucial role in recruiting downstream ATG proteins, while Double FYVE Domain Containing Protein 1 (DFCP1) promotes isolation membrane formation. P62 acts as an autophagy adaptor protein, binding to NCOA4 and ferritin to target them to the autophagosome. The ESCRT system assists in the closure of the autophagosome membrane during later stages. STX17 (Syntaxin 17) and SNAP29 (synaptosome associated protein 29) promote the fusion of autophagosomes with lysosomes, aided by Vesicle-Associated Membrane Protein (VAMP) seven and VAMP8 on the lysosome membrane. Ras-Related Protein 7 (Rab7) facilitates the maturation of autophagosomes and their fusion with lysosomes. Nuclear Factor Erythroid 2–Related Factor 2 (NRF2), a key regulator of oxidative stress, controls ferroptosis via HERC2 and VAMP8, influencing NCOA4 degradation and the fusion process. Disruption in ferritinophagy can lead to iron overload, generating reactive hydroxyl radicals, which can induce ferroptosis and disrupt cellular function.

Ferritinophagy is a specialized form of autophagy. It shares many similarities with the classical autophagy pathway, primarily involving three steps: the formation of autophagosomes, the fusion of autophagosomes with lysosomes, and the degradation and recycling of the contents (Figure 1). Ferritinophagy plays a crucial role in regulating cellular iron balance, ensuring that iron is properly utilized in metabolism. If ferritinophagy is impaired, it can lead to iron overload, where excess iron participates in the Fenton reaction. This generates reactive hydroxyl radicals (•OH), which are highly oxidative and can damage cell membranes, potentially triggering lipid peroxidation and ferroptosis, a form of cell death that can disrupt liver function (Qian et al., 2024; Szarka et al., 2021).

Ferritinophagy is regulated by several factors that help maintain iron balance in the body. IRPs, such as IRP1 and IRP2, play a central role in controlling iron uptake, storage, and usage. When iron is abundant, IRP1 binds to Fe-S clusters, losing its ability to regulate ferritin production. However, in low iron conditions, IRP1 binds to iron-responsive elements (IREs) in ferritin mRNA, reducing ferritin synthesis to limit iron storage (Tong and Rouault, 2007). In contrast, IRP2 does not have Fe-S clusters. When iron is sufficient, IRP2 is degraded via the FBXL5-mediated ubiquitination pathway. Iron deficiency affects the stability of FBXL5, thereby enhancing the binding of IRP2 to IREs (Zhang et al., 2014; Sanchez et al., 2011). Additionally, the expression of NCOA4 is adjusted according to intracellular iron levels. When iron is sufficient, HERC2 recognizes NCOA4 and degrades it via ubiquitination to prevent iron toxicity. When iron is deficient, the expression of NCOA4 increases to promote ferritinophagy, thereby releasing more iron ions (Mancias et al., 2015). Besides, there are two classic pathways that respond to external nutrients and energy: the mTOR signaling pathway and the AMPK pathway. The mTOR pathway remains active when nutrients are sufficient, inhibiting autophagy, while the AMPK pathway acts in the opposite manner (Bao et al., 2022).

Upon receiving activation signals, the ULK complex (including proteins like ULK1/2 and ATG13) recruits and activates the PI3K complex (composed of VPS34, ATG14L, VPS15, and Beclin1) to generate phosphatidylinositol-3-phosphate (PtdIns3P). PtdIns3P accumulates at the autophagy initiation site, the isolation membrane (IM), and recruits other effector proteins, such as DFCP1 and WIPI1/2 (23, 24). Among them, DFCP1 can promote the formation and expansion of the isolation membrane (Nähse et al., 2024), WIPI1/2 act as effector proteins of PtdIns3P and are crucial for recruiting downstream ATG proteins (Gaugel et al., 2012). WIPI2’s primary function is to recruit the ATG16L complex (ATG5-ATG12-ATG16L). The ATG16L complex acts as an E3 enzyme and facilitates the lipidation of microtubule-associated proteins 1A/1B light chain 3 (LC3), converting it to LC3-II. And LC3 is one of the most important markers in the autophagy process, it integrates into the IM like bricks, providing essential structural support for the formation of the autophagosome. Additionally, the ATG16L complex can help in the expansion and closure of the IM membrane by affecting membrane curvature (Wilson et al., 2014; Noda and Yoshimori, 2009; Nakatogawa, 2013). How is the ATG16L complex formed? The process is as follows: First, ATG12 is activated through a process that requires ATP and interacts with other proteins like ATG7 and ATG10. This activation is essential for the formation of the autophagosome. Subsequently, under the catalysis of ATG10, ATG12 is covalently linked to ATG5, forming the ATG12-ATG5 covalent complex. Finally, the ATG12-ATG5 complex binds with ATG16L1 to form the final complex (Mizushima, 2020). It is also noteworthy that before being acted upon by E3 ligase, LC3 must first be cleaved at its C-terminus by ATG4, exposing a glycine residue that can conjugate with phosphatidylethanolamine (PE). Subsequently, LC3 is activated by ATG7 (E1) and ATG3 (E2), and finally conjugates with PE (29, 31).

In addition to the proteins involved in forming the autophagic membrane, certain transport proteins are essential for providing the necessary lipids and other materials. ATG9 is a membrane protein that helps transfer lipids and proteins from organelles like the endoplasmic reticulum and Golgi apparatus to the site of autophagosome formation, ensuring the membrane can expand. ATG2 also aids in this process by facilitating lipid transfer between the endoplasmic reticulum and the autophagic membrane. Together, ATG2 and ATG18 help recruit lipids to the membrane, which is crucial for the expansion of the autophagosome (Obara et al., 2008; Osawa and Noda, 2019; He and Klionsky, 2007).

Ferritinophagy, as a specific form of autophagy, relies on the precise recognition of ferritin. NCOA4 is the key regulatory factor in this process. It binds to ferritin and directs it to the IM. Once the autophagosome matures and fuses with the lysosome, ferritin is degraded, releasing iron ions. These ions are then transported out of the cell by ferroportin1 (FPN1), participating in systemic iron metabolism. It is also important to note that intracellular iron uptake requires another transporter, Divalent Metal Transporter 1 (DMT1), and iron transport mediated by DMT1 is typically facilitated through the formation of endosomes (Yanatori and Kishi, 2019; Ajoolabady et al., 2021; Anderson and Frazer, 2017). In this process, P62/SQSTM1 acts as an autophagy adaptor protein, regulating NCOA4 and inducing the localization of the NCOA4-ferritin complex to the autophagosome (Liu et al., 2023). In the later stages of autophagy, the ESCRT system assists in the closure of the autophagosome membrane, enabling the autophagosome to mature into a complete vesicle (Lefebvre et al., 2018). STX17 and SNAP29, which are localized on the autophagosome, can also promote the fusion of autophagosomes and lysosomes by forming a complex with VAMP7/8, which is located on the lysosome (Tian et al., 2021). Meanwhile, Rab7 can also promote the maturation of autophagosomes and their fusion with lysosomes by interacting with effector proteins (Ao et al., 2014). The transcription factor nuclear factor erythroid 2–related factor 2 (NRF2) is primarily responsible for regulating the cellular antioxidant stress response. Under normal conditions, NRF2 binds to Keap1, which promotes NRF2 ubiquitination, thus maintaining its low levels within the cell. When cells are exposed to oxidative stress or toxic substances, the structure of Keap1 changes, allowing NRF2 to be released and translocate into the nucleus. Once in the nucleus, NRF2 binds to the antioxidant responsive element (ARE), initiating the transcription of antioxidant and detoxifying genes (Fan et al., 2017). Anandhan et al. discovered that NRF2 can regulate ferroptosis through HERC2 and VAMP8. HERC2 is an E3 ubiquitin ligase for NCOA4, facilitating its degradation, while VAMP8 plays a role in the fusion of autophagosomes and lysosomes (Anandhan et al., 2023).

### Ferritinophagy in biochemical factor-induced liver injury

With the rapid progress of urbanization and industrialization, heavy metal pollution has become an increasingly severe issue. These metals pose a significant threat to public health, as many can induce liver damage through ferritinophagy. For instance, cadmium (Cd), a high-risk heavy metal, is prominently used in industries such as metallurgy, mining, battery manufacturing, pigments, and plastics (Wang M. et al., 2021). Research has shown that Cd induces liver damage in a dose-dependent manner. The underlying mechanism is closely linked to the ER stress-ferritinophagy axis. Intervening with an ER stress inhibitor, such as GSK, can significantly inhibit ferritinophagy in cells exposed to Cd, thereby alleviating ferroptosis (He et al., 2022). In addition, As is also a common chemical pollutant. It poses serious health risks to humans through food chain transmission and bioaccumulation (Clemens and Ma, 2016). Although the toxicity of As is not as high as that of heavy metals like Cd, lead, or mercury, its widespread presence in the environment poses significant risks. As can enter the human body through drinking water, food, air, and occupational exposure, making it more frequently encountered than other heavy metals. Consequently, the Agency for Toxic Substances and Disease Registry (ATSDR) has placed arsenic at the top of its Substance Priority List (Shakya et al., 2023; Chen and Costa, 2021; Garbinski et al., 2019). Recent reports have increasingly highlighted the hepatotoxic effects of arsenic. Wu and colleagues have found that extended exposure to low levels of As compounds can result in liver fibrosis (Wu M. et al., 2023). Wang et al. found that long-term consumption of drinking water containing inorganic arsenic (iAs) increases the risk of liver cancer (Wang et al., 2014). Additionally, As has been shown to induce the accumulation of intracellular ROS and trigger ferroptosis (Gao et al., 2024). However, it remains unclear whether chronic As exposure induces ferroptosis by enhancing hepatic ferritinophagy. To investigate this, Yu et al. conducted a study and discovered that chronic arsenic exposure activates the AMPK/mTOR/ULK1 axis, triggering ferritinophagy-mediated ferroptosis and leading to liver damage (Yu L. et al., 2023). Another heavy metal commonly encountered in daily life is cuprum (Cu). The widespread misuse of cuprum-based fungicides and fertilizers in agriculture, along with the indiscriminate discharge of industrial wastewater, has led to increasing soil contamination with cuprum (Li et al., 2020). The hepatotoxic mechanisms of cuprum are complex and multifaceted. On one hand, cuprum induces oxidative stress through a mitochondrial-mediated caspase-dependent pathway (Yang et al., 2019), On the other hand, it can cause mitochondrial autophagy through the PINK1/Parkin pathway (Yang et al., 2021), Additionally, it promotes ferroptosis by inducing NCOA4-mediated ferritinophagy, inhibiting the antioxidant stress signaling pathway NRF2/Keap1, and suppressing Ferroptosis Suppressor Protein 1 (FSP1) (Zhong et al., 2024).

Silica nanoparticles (SiNPs) are inorganic materials with unique physicochemical properties. Due to these properties, they are widely used in various fields, including biomedicine, chemical engineering, pharmaceuticals, and cosmetics, playing a crucial role in modern technology (Huang et al., 2022; Wang et al., 2018). According to the ‘Guidelines on Protecting Workers from Potential Risks of Engineered Nanomaterials’ published by the World Health Organization, SiNPs are the second most produced engineered nanomaterials globally, with an annual production reaching up to 1.5 million tons (WHO Guidelines Approved by the Guidelines Review Committee, 2017). With the widespread use of SiNPs, the potential for human exposure has increased, drawing significant attention from researchers and regulatory agencies. Previous studies have demonstrated that SiNPs can induce autophagy in various cell lines (Ma et al., 2022; Zhu Y. et al., 2023). A 2018 study found that silica nanoparticles can mediate autophagy in hepatocytes through the EIF2AK3 and ATF6 UPR pathways (Wang et al., 2018). Building on this, Liang et al. found through both *in vivo* and *in vitro* experiments that prolonged exposure to SiNPs exacerbates liver fibrosis, with the underlying mechanism involving NCOA4-mediated ferritinophagy. Furthermore, upon cessation of SiNPs exposure, the activation of ferritinophagy decreased, thereby alleviating the severity of liver fibrosis (Liang et al., 2023). Deoxynivalenol (DON), also known as vomitoxin, is a mycotoxin produced by certain Fusarium species, particularly Fusarium graminearum and Fusarium culmorum. These fungi commonly infect essential cereal crops such as wheat, barley, rye, oats, and maize, posing a significant challenge to public food safety (Liao et al., 2018). The liver, being the most important detoxification organ in the body, is inevitably damaged by the metabolism of most mycotoxins. In 2020, Peng et al. demonstrated that prolonged exposure to DON impairs liver function, with molecular-level observations showing significant increases in both apoptosis and autophagy in hepatocytes (Peng et al., 2020). Jiang et al. further explored the connection between ferritinophagy and DON-induced liver injury. They found that DON promotes autophagy by activating the expression of mammalian target of rapamycin complex 1 (mTORC1). Additionally, DON enhances the phosphorylation of NCOA4 through ataxia-telangiectasia mutated kinase (ATM), increasing its affinity for ferritin and thereby promoting ferritinophagy and ferroptosis (Jiang et al., 2024a). Recent research has proposed a potential therapeutic approach for DON-induced liver injury: glycyrrhetinic acid (GA) can bind to programmed cell death protein 4 (PDCD4) to inhibit its ubiquitination and degradation. The stabilization of PDCD4 downregulates NCOA4 expression through the JNK-Jun-NCOA4 axis, thereby inhibiting ferritinophagy and effectively mitigating liver injury induced by DON(64).

### Ferritinophagy in drug-induced acute liver injury

Drug-Induced Liver Injury (DILI) is a common liver disease in clinical practice. In severe cases, it can lead to liver failure and even be life-threatening. Notably, in Western societies, DILI is the leading cause of acute liver failure (Katarey and Verma, 2016). Specifically, one of the major causes of DILI is acetaminophen (APAP) overdose. As one of the most widely used analgesic and anti-inflammatory drugs globally, APAP has been beneficial to patients around the world. Excessive consumption, on the other hand, can lead to severe liver damage (Ramachandran and Jaeschke, 2019). The exact mechanism by which APAP impacts liver function remains unclear. Existing research indicates that APAP produces a toxic metabolite, N-acetyl-p-benzoquinone imine (NAPQI). Normally, NAPQI is neutralized and rendered non-toxic by binding with glutathione (GSH). When NAPQI accumulates excessively in the liver, it leads to GSH depletion, contributing to liver damage (Nguyen and Stamper, 2017). GSH, as a crucial antioxidant in cells, plays a key role in preventing lipid peroxidation. When GSH is depleted, the levels of ROS in the cell increase, leading to lipid peroxidation and ultimately resulting in ferroptosis (Niu et al., 2021). Researchers have explored this issue by synthesizing a novel antioxidant enzyme nanomaterial—flower-shaped MnO₂ nanoparticles (MnO₂Nfs). These nanoparticles effectively improve acute liver injury induced by APAP. They not only possess various antioxidant enzyme activities to reduce ROS generation but also inhibit ferritinophagy by suppressing FTH/L degradation and LC3-II expression (Wu et al., 2024). Similarly, Shan and colleagues utilized manganese to create poly (acrylic) acid-coated Mn₃O₄ nanoparticles (PAA@Mn₃O₄-NPs, PMO). This material demonstrates antioxidant properties and good biocompatibility, allowing for liver accumulation and presence in lysosomes. By eliminating ROS, it inhibits ferritinophagy and counters acute liver injury induced by APAP (70).

Another noteworthy drug is methotrexate (MTX). As a folate antagonist, MTX inhibits dihydrofolate reductase (DHFR) to block the synthesis of DNA, RNA, and proteins. Low doses (5–25 mg/week) of MTX are first-line treatments for rheumatoid arthritis and other inflammatory joint diseases, while high doses, typically defined as 500 mg/m^2^, are commonly used to treat various malignant tumors (Pivovarov and Zipursky, 2019; Howard et al., 2016). However, MTX’s hepatotoxicity has long been a concern for clinicians. Although recent studies suggest that the risk of MTX-induced liver fibrosis might be overestimated (Andrade and Björnsson, 2023), there is broad consensus that long-term use of MTX can lead to liver damage (Ezhilarasan, 2021). Wang and colleagues investigated the mechanism of MTX-induced hepatotoxicity and found that MTX triggers ferritinophagy mediated by NCOA4 within liver cells through high-mobility group box 1 (HMGB1), ultimately leading to liver damage. Additionally, they discovered that GA, an HMGB1 inhibitor, can effectively alleviate MTX-induced liver injury (Wang C. et al., 2024).

Tuberculosis (TB) is a common infectious disease caused by *Mycobacterium* TB and is a leading cause of death from infectious diseases among adults worldwide. The standard treatment regimen typically involves a 6-month multi-drug therapy, which commonly includes isoniazid (INH), rifampin (RIF), pyrazinamide (PZA), and ethambutol (EMB) (Furin et al., 2019). During anti-TB treatment, one of the most frequent adverse reactions is anti-TB drug-induced liver injury. Studies have shown that RIF, INH, and PZA can induce hepatotoxicity through mitochondrial autophagy (Elmorsy et al., 2017). Zhou and colleagues analyzed RIF-induced liver toxicity from the perspective of ferritinophagy. They found that RIF exacerbates liver damage by inhibiting the expression of Human 71 kDa heat shock cognate protein (HSPA8), which increases ferritinophagy (Zhou et al., 2022).

### Ferritinophagy in liver fibrosis

Liver fibrosis is a pathological process in which normal liver tissue is gradually replaced by fibrous tissue (scar tissue) due to chronic injury. The development of liver fibrosis involves several key steps: 1. Chronic liver damage from various causes, such as hepatitis viruses, alcoholic hepatitis, and NAFLD. 2. Inflammatory cells release cytokines and chemical signals that promote the activation of HSCs. 3. Activated HSCs transform into myofibroblast-like cells and begin to synthesize and secrete large amounts of extracellular matrix (ECM), including collagen. 4. As fibrous tissue accumulates in the liver, the liver’s structure is progressively destroyed, leading to a decline in liver function (Aydın and Akçalı, 2018; Roehlen et al., 2020).

Previously, liver fibrosis was generally regarded as a passive and irreversible process. However, current understanding suggests that early-stage liver fibrosis can be slowed, arrested, or even reversed with prompt and suitable intervention. Once fibrosis advances to late-stage cirrhosis, reversal becomes highly challenging, and therapeutic options are significantly constrained. In such advanced cases, liver transplantation may be the only effective recourse (Aydın and Akçalı, 2018; Bataller and Brenner, 2005). The activation of HSCs is a hallmark event in liver fibrosis. Regulating HSC activation could enable precise antifibrotic therapies, offering the potential to effectively treat liver fibrosis (Higashi et al., 2017).

Kong et al. discovered that artesunate, a stable derivative of artemisinin, can promote ferritinophagy in activated HSCs *in vitro*, thereby inhibiting the progression of liver fibrosis. PCR results indicated that artesunate can enhance the expression of several ferritinophagy-related genes, including Atg3, Atg5, Atg6/beclin1, Atg12, and LC3 (17). Another compound, Oroxylin A (OA), extracted from the traditional Chinese herb Scutellaria baicalensis, was shown by Sun and colleagues to mediate ferritinophagy through the cGAS-STING pathway, inducing HSC senescence and ultimately inhibiting liver fibrosis. In their experiments, they found that increasing doses of OA led to a corresponding increase in NCOA4 expression, while the expression of FTH1 decreased. FTH is a crucial protein responsible for storing and regulating iron ions within cells. A decrease in FTH1 implies an increase in intracellular free iron ions, which typically leads to increased ferritinophagy. Additionally, autophagy markers and initiators such as LC3 and Beclin1 showed dose-dependent expression increases. This indicates that OA can induce ferritinophagy in HSCs. Furthermore, when HSCs were treated with NCOA4 siRNA, the expression of senescence markers (p16, p21, and HMGA1) was significantly downregulated compared to the control group, leading to the conclusion that OA regulates HSC senescence through ferritinophagy. Subsequently, using cGAS siRNA to inhibit the cGAS-STING pathway in HSCs resulted in decreased expression of NCOA4, LC3, and Beclin1, along with reduced intracellular ROS levels and iron ion content. This demonstrated that the effect of OA on ferritinophagy in HSCs is regulated by the cGAS-STING pathway (Sun et al., 2023). Schisandra chinensis, another traditional Chinese herb, has recently been found to possess various biological activities, including antioxidant, anti-inflammatory, hepatoprotective, and anti-tumor properties (Zhang W. et al., 2020). Recent studies have shown that Schisandrin B, an active compound extracted from Schisandra chinensis, inhibits the activation of HSCs and induces macrophage polarization, thereby alleviating liver fibrosis (Chen et al., 2017; Chen et al., 2021). Ma et al. studied Schisandrin B from the perspective of HSCs senescence. They found that Schisandrin B can regulate ferritinophagy mediated by NCOA4 in HSCs, inducing iron overload and subsequent oxidative stress. This promotes the senescence of activated HSCs, ultimately improving the degree of liver fibrosis (Ma et al., 2023). Taurine, one of the most abundant amino acids in mammals, has a protective effect against liver fibrosis caused by cellular oxidative stress (Miyazaki et al., 2005). Although Li et al. did not conduct NCOA4 knockout and overexpression experiments to confirm the relationship between taurine and ferritinophagy, their bioinformatics analysis revealed a strong molecular docking interaction between taurine and NCOA4, suggesting that taurine may potentially target NCOA4-mediated ferritinophagy, thereby promoting ferroptosis in HSCs and reducing ECM deposition (Li et al., 2024). Future researchers could use this as a starting point to further investigate and elucidate the underlying mechanisms.

In addition to the natural compounds mentioned above, research in molecular biology has also explored this area. BECN1 was discovered as early as 2018 to promote lipid peroxidation and iron death by inhibiting the important regulatory system of iron death, system xc−(90). Zhang et al. found that the RNA-binding protein ELAVL1/HuR can bind to the 3′untranslated region of BECN1 mRNA, increasing its stability and thereby activating ferritinophagy to induce ferroptosis in HSCs. Additionally, sorafenib (SF) has been shown to upregulate ELAVL1 expression, which alleviates the severity of liver fibrosis. This suggests a potential therapeutic pathway where SF modulates ferroptosis-related pathways to mitigate liver damage (Zhang et al., 2018). Mesenchymal Stem Cells (MSCs) are recognized for their pluripotency, immune regulation, and self-renewal capabilities, which suggest their potential clinical application value. Previous studies have demonstrated that MSCs and their derived exosomes (MSC-ex) have shown considerable abilities in inhibiting liver fibrosis, acute liver injury, and acute liver failure (Shokravi et al., 2022; Shao et al., 2020; Li et al., 2013). After a deeper understanding of MSCs’ mechanisms, Tan and colleagues found that MSC-ex promotes the expression of BECN1 to increase intracellular LC3 and Fe2^+^ levels, thereby inducing ferritinophagy and promoting HSCs iron death to reduce liver fibrosis (Tan et al., 2022). On the other hand, another RNA-binding protein, ZFP36/TTP, exhibits a contrasting role. It binds to ATG16L1 mRNA, promoting its degradation and thus leading to inactivation of ferritinophagy in HSCs and resulting in resistance to ferroptosis (Zhang Z. et al., 2020).

### Ferritinophagy in liver cancer

HCC is the most common type of primary liver cancer. According to the Global Cancer Statistics 2020, HCC ranks sixth in global cancer incidence and third in cancer-related mortality (Sung et al., 2021). While SF is a first-line treatment for advanced HCC, it offers limited improvement in patient survival, and many HCC patients develop adaptive resistance to SF(98, 99). Therefore, finding suitable adjunctive drugs to enhance the efficacy of SF may be highly beneficial for HCC patients.

Caryophyllene Oxide, a naturally occurring sesquiterpene compound, possesses various biological activities. Historically used in cosmetics and food additives for its unique aroma, recent studies have highlighted its significant antitumor and analgesic properties (Fidyt et al., 2016; Kim et al., 2014). Research in 2019 demonstrated that β-Caryophyllene Oxide could indirectly increase the sensitivity of HCC to SF, enhancing its cytotoxic effects (Di Giacomo et al., 2019). Further investigation by Xiu and colleagues explored the association between Caryophyllene Oxide and ferritinophagy in HCC cells. They found that Caryophyllene Oxide could trigger ferritinophagy and mediate ferroptosis in HCC cells by increasing the expression levels of NCOA4 and LC3Ⅱ, while decreasing FTH1 expression. This led to a significant reduction in liver tumor volume, suggesting that Caryophyllene Oxide could be a potential therapeutic agent for HCC(103). Additionally, Artesunate, recognized for its ability to induce ferritinophagy, is considered an ideal adjunctive therapy. Li and colleagues investigated the combined effects of Artesunate and SF on HCC cells, finding that the combination treatment enhanced mitochondrial damage, lysosomal activation, and ferritinophagy, ultimately leading to ferroptosis. These findings highlight the potential value of the synergistic effects between artemisinin derivatives and SF in HCC treatment, suggesting that this combination could offer an innovative and clinically promising strategy for managing HCC(104).

Esculetin, a natural dihydroxycoumarin commonly derived from the bark of Fraxinus chinensis Roxb, has garnered significant research interest due to its diverse pharmacological activities, including antioxidant, anti-inflammatory, antitumor, and antibacterial effects (Garg et al., 2022). As early as 2015, studies demonstrated that esculetin could induce ROS accumulation and apoptosis in gastric cancer cells through a caspase-dependent pathway, achieving its antitumor effects (Pan et al., 2015). However, research on esculetin’s role in inducing ferritinophagy in HCC cells is limited. Xiu et al. investigated this aspect and found that, similar to Caryophyllene Oxide, esculetin can regulate ferritinophagy through the NCOA4/LC3-II/FTH1 signaling pathway. This regulation helps suppress HCC cell proliferation and differentiation (Xiu et al., 2023). Eupatorium chinense L., a traditional Chinese medicinal herb, is known for its antitumor, antibacterial, and anti-inflammatory properties. Zhu and colleagues extracted a sesquiterpene from Eupatorium chinense L. and discovered that this compound can upregulate NCOA4 expression to mediate ferritinophagy and disrupt mitochondrial function, thereby enhancing HCC cell apoptosis (Zhu ZH. et al., 2023).

Research from the molecular biology perspective has provided valuable insights into the treatments of liver cancer. Polypyrimidine Tract Binding Protein 1 (PTBP1) is a crucial RNA-binding protein with significant regulatory roles across various systems, including the nervous, immune, and cardiovascular systems, as well as in tumors (Yu Q. et al., 2023). Yang and colleagues discovered that PTBP1 can promote ferritinophagy in HCC cells by binding to NCOA4 mRNA, which enhances the sensitivity of HCC cells to ferroptosis following SF treatment (Yang et al., 2023). Additionally, Wang and colleagues found that in HCC cell models, activators of ferroptosis (such as SF, erastin, and sulfasalazine) induce ferritinophagy, leading to the activation of AMPK phosphorylation. BCAT2 is a key enzyme in glutamate production, and the function of system Xc–is directly regulated by glutamate levels. AMPK/SREBP1 signaling pathway inhibits the nuclear translocation of the transcription factor SREBP1 and suppresses the transcription of BCAT2. Inhibition of BCAT2 results in decreased intracellular glutamate, causing reduced cystine uptake and thereby inducing ferroptosis in tumor cells (Wang K. et al., 2021).

### Ferritinophagy in Non-Alcoholic fatty liver disease

Non-Alcoholic Fatty Liver Disease (NAFLD) is characterized by the accumulation of fat in the liver without the involvement of excessive alcohol consumption. As one of the most prevalent chronic liver diseases worldwide, NAFLD’s high prevalence is closely linked to modern dietary and lifestyle habits. Metabolic syndrome (MS) is a key risk factor for NAFLD, encompassing conditions such as obesity, hypertension, hyperglycemia, and hyperlipidemia (Friedman et al., 2018; Huang, 2009). Research and epidemiological studies have demonstrated that dysregulation of iron homeostasis, leading to iron overload, plays a significant role in the development of NAFLD (114). Kobi et al. discovered that iron overload can promote the nuclear translocation of transcription factor EB (TFEB) in NAFLD, which enhances lysosomal expression and increases the occurrence of ferritinophagy, and ultimately aggravating the severity of disease (Honma et al., 2023). Ferritinophagy plays a critical role in releasing iron from ferritin stores, aiding in the oxidative desaturation of fatty acids and the synthesis of triglycerides and cardiolipins, which helps mitigate lipotoxicity. This process plays a critical role in cellular metabolism, ensuring that iron is available for key biochemical pathways. While beneficial in the short term by preventing the accumulation of toxic lipid intermediates, chronic induction of ferritinophagy can have detrimental effects. Over time, the sustained mobilization of iron may lead to excessive iron accumulation in the liver, promoting oxidative stress and inflammation. This contributes to the development of hepatic steatosis, a condition characterized by the abnormal retention of fat in liver cells, and can progress to more severe liver diseases such as non-alcoholic steatohepatitis (NASH), fibrosis, and ultimately cirrhosis. Therefore, while ferritinophagy is a vital adaptive response to metabolic stress, its persistent activation underscores the delicate balance required to maintain liver health and prevent long-term hepatic damage (Li et al., 2022).

Insulin resistance, characterized by the reduced sensitivity of the body to insulin and the diminished ability to promote glucose uptake and utilization (Samuel and Shulman, 2016), is a common feature of metabolic disorders. The prevailing view is that insulin resistance and the resulting hyperinsulinemia are key factors in the pathogenesis of NAFLD (117). Prolonged high-fat diets have been found to inhibit ferritinophagy, disrupting iron homeostasis, leading to endoplasmic reticulum stress, and ultimately mediating the development of insulin resistance. This may explain why individuals who excessively consume high-fat foods are more prone to NAFLD (118). Chronic intermittent hypobaric hypoxia (CIHH) refers to a treatment method that induces adaptation and enhances tolerance to hypoxic environments through intermittent exposure to low oxygen conditions. Although CIHH has shown potential in some studies and experiments, its widespread clinical use requires further research and validation (Zhang et al., 2020c). Cui and colleagues discovered a link between CIHH and ferritinophagy. They found that CIHH treatment in MS rats upregulated the expression of NCOA4, promoting ferritinophagy and the expression of FPN, thereby reducing oxidative stress and iron overload. This ultimately inhibited hepatocyte ferroptosis and alleviated NAFLD (114). Insulin Growth Factor Binding Protein 7 (IGFBP7) is a protein that regulates the activity of insulin-like growth factors (IGF), which are crucial for cell growth, differentiation, and metabolism. IGFBP7 modulates the biological functions of IGF by binding to it (Jin et al., 2020). Previous studies have found that IGFBP7 exacerbates hepatic steatosis in the development of NAFLD, although the exact mechanisms remain unclear (Yan et al., 2019). Wang and colleagues conducted further research and discovered that IGFBP7 promotes the progression of NAFLD by inducing ferroptosis through NCOA4-mediated ferritinophagy. Depletion of IGFBP7 effectively improved liver inflammation, fibrosis, and steatosis (Wang et al., 2023).

### Ferritinophagy in sepsis-associated liver injury

Sepsis is characterized by a systemic infection and a strong inflammatory response, potentially leading to multiple organ dysfunction or failure, and even death. The liver, as a critical metabolic and detoxification organ, is a primary target of sepsis-associated damage. Liver dysfunction frequently occurs in the early stages of sepsis and is often considered a marker of poor prognosis. Understanding the underlying mechanisms of sepsis-induced liver injury is essential for developing effective therapeutic strategies (Yan et al., 2014).

YAP1 (Yes-associated protein 1) and TAZ (WW domain-containing transcription regulator 1) are important downstream effectors of the Hippo signaling pathway, which regulates gene expression through transcription factors. The Hippo/YAP1 pathway plays significant roles in tissue growth, organ size regulation, and cancer development (Panciera et al., 2017). Additionally, there is evidence that YAP1 has positive effects in liver-related diseases, such as promoting liver regeneration and repair and inhibiting liver fibrosis (Liu et al., 2019; Wang et al., 2020). Wang et al. discovered that in a sepsis-induced liver injury model, YAP1 can inhibit NCOA4-mediated ferritinophagy, thereby suppressing the expression of SFXN1 (a mitochondrial membrane iron transport protein). This inhibition prevents ferroptosis in hepatocytes and effectively alleviates liver damage (Wang et al., 2022). Similarly, Qi et al. observed comparable effects in a NAFLD model. They found that curcumol can reduce NCOA4 expression through YAP, regulating ferritinophagy and ultimately preventing hepatocyte senescence (Qi et al., 2021). There are few studies on ferritinophagy and sepsis-associated liver injury, but based on literature review, I believe the following targets may be related to ferritinophagy: GPR116, an adhesion G protein-coupled receptor (GPCR), can inhibit key ferroptosis targets such as system Xc, GSH, and GPX4, inducing sepsis-related liver injury (Yan et al., 2014). GLI Family Zinc Finger 2 (GLI2) is a key regulator in the Hedgehog (Hh) signaling pathway, which plays an important role in tissue development during embryogenesis and is thought to promote liver regeneration (Omenetti et al., 2011), Sun et al. found that GLI2 exacerbates sepsis-related liver injury by regulating the expression of synovial apoptosis inhibitor 1 (SYVN1), which inhibits PPARα-mediated autophagy (Sun et al., 2024). Hypoxia-Inducible Factor 1-Alpha (HIF-1α) is a transcription factor induced under hypoxic conditions. Several studies have shown that HIF-1α can inhibit ferritinophagy under hypoxia, protecting cells from ferroptosis (Ni et al., 2021; Wang Y. et al., 2024). In sepsis-associated liver injury, Zhu et al. discovered that fibroblast growth factor 21 (FGF21) can promote HIF-1α expression, effectively suppressing macrophage activation and reducing inflammation after liver injury. However, whether ferritinophagy is involved in this process requires further investigation (Zhu et al., 2024). While analyzing recurrently dysregulated genes in sepsis, Deng et al. discovered that the long non-coding RNA Mir22 hg contributes to ferritinophagy-mediated ferroptosis by recruiting the m6A reader YTHDC1 and stabilizing Angptl4 mRNA. This finding suggests that Mir22 hg could serve as a potential therapeutic target for treating sepsis through the modulation of ferroptosis (Deng et al., 2024).

At the end of this article, we provide a summary of the role of ferritinophagy in various liver diseases, as well as its regulatory factors. To facilitate comprehension, we have compiled (Table 1), which lists factors that can exacerbate liver diseases through ferritinophagy related pathways, and (Table 2), which lists factors that may ameliorate liver diseases through these pathways.

**TABLE 1:** Regulatory core substances participate in the aggravation of liver diseases by regulating ferritinophagy.

Substance	Mechanism	Effect (positive or negative)	Disease	References
Cd	ER Stress-Ferritinophagy Axis	Negative	biochemical factor-induced liver injury	He et al. (2022)
As	AMPK/mTOR/ULK1 Axis Activation by Chronic Arsenic Exposure	Negative	biochemical factor-induced liver injury	Yu et al. (2023a)
Cu	Mitophagy, NCOA4 Induction, Nrf2/Keap1 and FSP1 Pathway	Negative	biochemical factor-induced liver injury	Zhong et al. (2024)
SiNPs	NCOA4-Mediated Ferritinophagy	Negative	biochemical factor-induced liver injury	Liang et al. (2023)
DON	Activates mTORC1, Promoting NCOA4 Phosphorylation	Negative	biochemical factor-induced liver injury	Jiang et al. (2024a)
MTX	Induces NCOA4 through HMGB1	Negative	Drug-Induced Liver Injury	Wang et al. (2024a)
RIF	HSPA8 Inhibition Increases Ferritinophagy	Negative	Drug-Induced Liver Injury	Zhou et al. (2022)
ZFP36/TTP	Promotes ATG16L1 mRNA Decay, Leading to Ferritinophagy Inactivation in HSCs	Negative	liver fibrosis	Zhang et al. (2020b)
MS	Iron Overload Promotes Nuclear Translocation of TFEB, Enhancing Lysosome Expression and Ferritinophagy	Negative	NAFLD	Honma et al. (2023)
high-fat diets	Inhibition of Ferritinophagy Disrupts Iron Homeostasis, Induces ER Stress, and Mediates Insulin Resistance	Negative	NAFLD	Jiang et al. (2020)
IGFBP7	Ferroptosis Induction via NCOA4-Mediated Ferritinophagy	Negative	NAFLD	Wang et al. (2023)

**TABLE 2:** Regulatory core substances participate in the improvement of liver diseases by regulating ferritinophagy.

Substance	Mechanism	Effect (positive or negative)	Disease	References
GA	Inhibits PDCD4 Ubiquitination, Leading to Downregulation of NCOA4 via the JNK-Jun-NCOA4 Axis	Positive	biochemical factor-induced liver injury	Jiang et al. (2024b)
MnO_2_Nfs	Prevents FTH/L Degradation, Inhibits LC3-II Expression, and Provides Antioxidant Protection	Positive	Drug-Induced Liver Injury	Wu et al. (2024)
PAA@Mn_3_O_4_-NPs	Antioxidant Activity and Inhibition of ROS generation	Positive	Drug-Induced Liver Injury	Shan et al. (2023)
GA	Acts as an HMGB1 inhibitor	Positive	Drug-Induced Liver Injury	Wang et al. (2024a)
artesunate	Enhances Ferritinophagy Gene Expression in HSCs, Such as Atg3, Atg5, Atg6/Beclin1, Atg12, and LC3	Positive	Liver fibrosis	Kong et al. (2019)
OA	Induces HSC Senescence through cGAS-STING-Mediated Ferritinophagy	Positive	Liver fibrosis	Sun et al. (2023)
Schisandrin B	Regulates NCOA4 to Mediate Ferritinophagy in HSCs	Positive	Liver fibrosis	Ma et al. (2023)
taurine	Exhibits Strong Molecular Docking Interaction with NCOA4	Positive	liver fibrosis	Li et al. (2024)
ELAVL1/HuR	Binds to the 3′UTR of BECN1 mRNA, Enhancing Stability and Activating Ferritinophagy in HSCs	Positive	Liver fibrosis	Zhang et al. (2018)
MSC-ex	Promotes BECN1 Expression to Enhance Intracellular LC3 and Fe2+ Levels	Positive	Liver fibrosis	Tan et al. (2022)
Caryophyllene Oxide	Promotes Ferritinophagy in Tumor Cells through Increased NCOA4 and LC3II Expression and Decreased FTH1 Levels	Positive	HCC	Xiu et al. (2022)
artesunate	Activates Lysosomes to Mediate Ferritinophagy	Positive	HCC	Li et al. (2021)
esculetin	Regulates Ferritinophagy via the NCOA4/LC3II/FTH1 Signaling Pathway	Positive	HCC	Xiu et al. (2023)
Eupatorium chinense L	Increases NCOA4 Expression, Mediating Ferritinophagy and Disrupting Mitochondrial Function	Positive	HCC	Zhu et al. (2023b)
PTBP1	Promotes Ferritinophagy in HCC Cells by Binding to NCOA4 mRNA	Positive	HCC	Yang et al. (2023)
Ferroptosis activators (SF, erastin, and sulfasalazine)	Activates AMPK/SREBP1 Signaling by Regulating Ferritinophagy, Inhibiting BCAT2 Transcription	Positive	HCC	Wang et al. (2021b)
CIHH	Upregulates NCOA4 and FPN to Promote Ferritinophagy and Iron Export, Mitigating Liver Injury	Positive	NAFLD	Cui et al. (2023)
YAP1	Inhibits NCOA4-Mediated Ferritinophagy and SFXN1 Expression, Reducing Ferroptosis	Positive	Sepsis-associated liver injury	Wang et al. (2022)
curcumol	Regulates Ferritinophagy by Reducing NCOA4 Expression via YAP, Preventing Hepatocyte Senescence	Positive	NAFLD	Qi et al. (2021)

### Interactions of ferritinophagy with other forms of selective autophagy

Ferritinophagy is not an isolated process, it interacts with other forms of selective autophagy, such as mitophagy (mitochondrial autophagy) and lipophagy (lipid droplet autophagy). These interactions play a critical role in maintaining cellular homeostasis and can significantly impact disease progression. Under conditions of oxidative stress, ferritinophagy is upregulated to release iron for essential metabolic processes. However, excessive iron release, through the Fenton reaction, leads to the generation of ROS, which in turn causes a reduction in mitochondrial membrane potential and mitochondrial dysfunction. This dysfunction activates the PINK1/Parkin signaling pathway to inhibit mitophagy (Zhang et al., 2020d). Furthermore, iron overload activates autophagy-related signaling pathways by generating ROS and increasing intracellular calcium levels. Concurrently, lipophagy, mediated by specific receptors such as Rab10 and DNM2, promotes ferroptosis, thereby exacerbating the pathological progression of non-alcoholic steatohepatitis (Honma et al., 2023). Understanding the complex crosstalk between ferritinophagy and other autophagy pathways is essential for the development of comprehensive therapeutic strategies. Future research should focus on elucidating these interactions and exploring the potential for combined therapeutic approaches targeting multiple autophagy pathways to better manage NASH and related diseases.

## Discussion on the clinical application prospects of traditional Chinese medicines

Several Traditional Chinese Medicines (TCMs) have been reported to modulate ferritinophagy, suggesting their potential therapeutic applications in liver diseases. For example, artesunate (a derivative of artemisinin) and Oroxylin A (extracted from Scutellaria baicalensis) have been shown to induce ferritinophagy in HSCs, thereby mitigating liver fibrosis. Another compound, Schisandrin B from Schisandra chinensis, has demonstrated potential in regulating ferritinophagy and alleviating liver fibrosis. While these compounds show promise in preclinical studies, none have been specifically approved for clinical use targeting ferritinophagy.

Future directions for drug discovery:

1. Target Identification and Validation: Future research should focus on identifying specific TCM compounds that modulate ferritinophagy and elucidate their mechanisms of action.
2. Clinical Trials: Conducting well-designed clinical trials to assess the safety and efficacy of TCMs in modulating ferritinophagy is essential for their approval and integration into clinical practice.
3. Combination Therapies: Exploring the combination of TCMs with conventional therapies (e.g., for cancer or fibrosis) may enhance therapeutic outcomes by targeting multiple pathways, including ferritinophagy.
4. Biomarker Development: Developing biomarkers to monitor ferritinophagy activity in clinical settings could facilitate the evaluation of TCM efficacy and guide personalized treatment strategies.

## Conclusion

Ferritinophagy has garnered significant attention for its pivotal role in liver health and disease, influencing not only iron metabolism and cellular homeostasis but also various pathological liver conditions. Abnormal activation or inhibition of ferritinophagy is strongly linked to liver damage, fibrosis, and the development of cancer. Therefore, a comprehensive understanding of the mechanisms underlying ferritinophagy and its involvement in liver diseases is critical for the development of novel therapeutic strategies. Future research must focus on precisely regulating ferritinophagy to offer new insights into the prevention and treatment of liver diseases.

Despite promising findings in animal models, translating these results to clinical applications for human patients presents several challenges. First, significant physiological and pathological differences exist between animal models and humans. Liver metabolic pathways, the regulation of ferritinophagy, and drug responses can vary across species, meaning that dosages, mechanisms of action, and therapeutic effects effective in animals may not directly apply to humans. Moreover, human diseases are far more complex and heterogeneous than those observed in animal models. For example, the pathogenesis of NAFLD involves a range of factors, including genetic background, lifestyle, and metabolic syndrome components, which are difficult to fully replicate in animal models. Even when modulation of ferritinophagy shows positive effects in animals, the same therapeutic strategy may yield varying results in human patients due to individual variations.

Furthermore, drug safety and side effects pose critical challenges in the translational process. Drugs that appear safe in animal studies may lead to unpredictable side effects in human clinical trials, as they may interfere with other essential cellular processes while regulating ferritinophagy. This underscores the need for comprehensive toxicological evaluations in preclinical studies and close monitoring of patient responses during clinical trials.

Clinical trial design and execution also present significant hurdles. Recruiting the appropriate patient population, defining valid treatment endpoints, and ensuring ethical and scientific integrity require careful planning. For instance, in liver cancer trials, factors such as tumor stage, treatment history, and comorbidities must be considered to ensure the reliability of results.

In summary, while compelling evidence from biological models supports the potential role of ferritinophagy in liver disease treatment, substantial obstacles remain in translating these findings to human clinical practice. Ongoing research must continue to explore the mechanisms and regulation of ferritinophagy and its implications for human liver diseases, with a focus on optimizing clinical trial designs and ensuring the safety and efficacy of treatments for human patients.
